# Comparative Study on Pharmacokinetics of Four Long-Acting Injectable Formulations of Enrofloxacin in Pigs

**DOI:** 10.3389/fvets.2020.604628

**Published:** 2021-01-26

**Authors:** Salah Uddin Ahmad, Jichao Sun, Fusheng Cheng, Bing Li, Safia Arbab, Xuzheng Zhou, Jiyu Zhang

**Affiliations:** ^1^Key Laboratory of Veterinary Pharmaceutical Development, Ministry of Agriculture, Lanzhou, China; ^2^Key Laboratory of New Animal Drug Project of Gansu Province, Lanzhou, China; ^3^Lanzhou Institute of Husbandry and Pharmaceutical Sciences of Chinese Academy of Agricultural Sciences, Lanzhou, China; ^4^Faculty of Veterinary, Animal and Biomedical Sciences, Sylhet Agricultural University, Sylhet, Bangladesh

**Keywords:** pharmacokinetics, long-acting, enrofloxacin, HPLC, pig

## Abstract

A comparative study on pharmacokinetics of four long-acting enrofloxacin injectable formulations was investigated in 36 healthy pigs after intramuscular injection according to the recommended single dose @ 2.5 mg/kg body weight. The drug concentrations in the plasma were computed using high-performance liquid chromatography (HPLC) with fluorescence detection. WinNonLin5.2.1 software was used to analyze the experimental data and compared it under one-way ANOVA using SPSS software with a 95% confidence interval (CI). The main pharmacokinetic parameters, that is, the maximum plasma concentrations (C_max_), the time to maximum concentration (T_max_), area under the time curve concentration (AUC_all_) and Terminal half-life (T_1/2_) were 733.84 ± 129.87, 917.00 ± 240.13, 694.84 ± 163.49, 621.98 ± 227.25 ng/ml, 2.19 ± 0.0.66, 1.50 ± 0.37, 2.89 ± 0.24, 0.34 ± 0.13 h, 7754.43 ± 2887.16, 8084.11 ± 1543.98, 7369.42 ± 2334.99, 4194.10 ± 1186.62 ng h/ml, 10.48 ± 2.72, 10.37 ± 2.38, 10.20 ± 2.81, and 10.61 ± 0.86 h for 10% enrofloxacin (Alkali), 20% enrofloxacin (Acidic), Yangkang and control drug Nuokang® respectively. There were significant differences among C_max_, T_max_, and AUC_all_ of three formulations compare with that of the reference formulation. No significant differences were observed among the T_1/2_ for tested formulations compare with the reference formulation. The pharmacokinetic parameters showed that the tested formulations were somewhat better compared to the reference one. The calculated PK/PD indices were effective for bacteria such as *Actinobacillus pleuropneumoniae* and *Pasteurella multocida* with values higher than the cut-off points (C_max_/MIC_90_≥10–12 and AUC/MIC_90_ ≥ 125). However, they were not effective against bacteria like *Haemophilus parasuis, Streptococcus suis, E. coli*, and *Bordetella bronchiseptica* where lower values were obtained.

## Introduction

Fluoroquinolones are essential therapeutic agents used for the treatment of animal infection (1) against a wide variety of microorganisms, including Mycoplasma species (2). Enrofloxacin (ENR) is a third-generation fluoroquinolone having broad-spectrum antimicrobial activity used in the veterinary field (3) that enhances the possibility of selecting resisting bacteria (4). They are widely used in farm animals because of their antimicrobial activity against a wide range of pathogens, favorable pharmacokinetic properties, and little toxicity (5). Quinolones are not allowed to use in poultry, where eggs are consumed by humans (6). A documentary at the University of Georgia showed enrofloxacin sensitivity for gram-negatives was 89%, and gram-positive was 38% (7). Enrofloxacin is signified for the treatment of respiratory and alimentary tract infections (8). Enrofloxacin is accepted for the treatment and control of swine respiratory disease caused by *Pasteurella multocida, Streptococcus suis, Actinobacillus pleuropneumoniae*, and *Haemophilus parasuis* (9).

The most common diseases encountered by swine pathogens are porcine pleuropneumonia caused by *Actinobacillus pleuropneumoniae* (10); septicemia, colibacillosis and edema caused by *Escherichia coli* (11); meningitis, pneumonia, arthritis, and septicemia encountered by *Streptococcus suis* (12); exudative epidermitis caused by *Staphylococcus hyicus* (13); atrophic rhinitis and bronchopneumonia caused by *Bordetella bronchiseptica* (14). *S. suis* is also known as a zoonotic pathogen and may cause severe diseases in humans, such as endocarditis, septicemia, meningitis, sensorineural hearing loss, and arthritis (15).

After administration, enrofloxacin partially metabolizes into ciprofloxacin (CIP) in some species including pigs (16). The metabolic conversion of enrofloxacin to ciprofloxacin varies in different animal species, that is, 59% in dairy cows (17), 36% in sheep (18), 47% in buffalo calves (19), 64% in beef steers (17), and 51.5% in healthy pigs (16). CIP is also effective against Gram-positive, negative, aerobes, and mycoplasmas. Although CIP has limited usage in the veterinary field, as a metabolite of ENR in animals, it reduces animal mortality and enhances growth (20, 21). Enrofloxacin shows moderate bioavailability, reasonable protein binding, and better binding to tissues (4, 22). Although ENR is the classical antibiotics of the quinolone family, the misuse and overuse of antibiotics have been recognized as a significant cause of developing resistant pathogens (23) and may have adverse effects on human health from its residues in animals, such as allergy and ENR-resistant strains (20).

The long-acting enrofloxacin injection for livestock and poultry optimizes various components. It does not affect the curative effect while reducing the total dose, the treatment cost, and has the advantages of long-lasting effects, high-efficiency, safe and reliable preparation. Long-acting injections of enrofloxacin are suitable for various infectious diseases in the respiratory system, digestive system, urinary system, and soft tissues of the skin caused by susceptible bacteria and mycoplasmas (24).

Generally, PK/PD modeling is used to assess the clinical efficacy of antimicrobial agents (25). The most commonly used PK parameters are the maximum plasma concentration (C_max_) and the area under the plasma concentration-time curve (AUC) (26). Fluoroquinolones are known as concentration-dependent drugs, and AUC/MIC and C_max_/MIC are better interpreters for the antibacterial effect (27, 28). Therefore, it is crucial to calculate the C_max_ and AUC value of enrofloxacin against the pathogens in swine.

Our study aimed to investigate the pharmacokinetic profiles of four long-acting ENR injectable formulations in pigs after intramuscular administration at a single dose of 2.5 mg per kg body weight. To establish the safe and effective therapeutic management of drugs in pigs, this pharmacokinetics study will establish appropriate clinical treatment for the new formulations of enrofloxacin injection for farm animals, especially for pigs.

## Materials and Methods

### Drugs and Reagents

Enrofloxacin (Purity HPLC ≥ 98%), the reference standard was purchased from Shanghai Yuanye Bio-Technology Co., Ltd. (RTECS number: vb1993650, CAS registry number 93106-60-6). Ten percent enrofloxacin injection (alkali preparation, purity 98.27%, compositions: enrofloxacin hydrochloride 10 gm, hydroxypropyl β cyclodextrin 30 gm, sodium thiosulfate 0.2 gm, an appropriate amount of sodium hydroxide, and water up to 100 mL), 20% enrofloxacin injection (acidic preparation, purity 101.80%, compositions: enrofloxacin 20 gm, propylene glycol 30 mL, hydroxypropyl β cyclodextrin 40 gm, sodium bisulfite 0.2 gm, EDTA-2Na 0.01 gm, glacial acetic acid about 5 ml, and water up to 100 ml), and 10% enrofloxacin injection (mild alkali preparation, purity 101.44%, trade name: Yangkang, compositions: enrofloxacin 10 gm, arginine 15 gm, and water up to 100 ml) were provided by Shandong Dezhou Shenniu Pharmaceutical Co., Ltd. The control drug 20% enrofloxacin injection (mild acidic preparation, purity 98.44%, trade name: Nuokang®) was provided by Tianjin Zhongsheng Tiaozhan Biotechnology Co., Ltd. Ciprofloxacin (Lot No. 522D021, CAS. 85721-33-1) was purchased from National Institutes for Food and Drug Control, China. Acetonitrile (HPLC grade) was obtained from Fisher Scientific, Fair Lawn, NJ, USA. Methanol (HPLC grade) was obtained from Fisher Scientific Company, 112 Colonnade Road, Ottawa ON K2E 7L6, Canada. Formic Acid was obtained from Shanghai Aladdin Biochemical Technology Co. Ltd. No 196 Xinjinqiao Road, Pudong, Shanghai, China. Antibiotic-free standard plasma was purchased from Guangzhou Hongquan Bio-Technology Co. Ltd, China. Watson's water was used for HPLC and to prepare 0.1% formic acid.

### Methodology Establishment

Established a sensitive, specific, accurate, and reliable method for quantitative analysis of biological samples and confirmed the method. In this study, the protein was precipitated by methanol to establish an HPLC method for the determination of ENR in pig plasma.

### Standard Solution Preparation

First stock solution of 1 mg/ml ENR was prepared by adding 25 mg ENR into 25 ml acetonitrile (HPLC Grade) in a 25 ml capacity volumetric flask. The stock solution was stored at 4°C for the preparation of 2^nd^ stock solutions and further use. Second stock solutions of ENR of 500 μg/ml, 50μg/ml, 5 μg/ml, and 1 μg/ml were made by adding the required amount of mobile phase. The same procedure was followed to prepare the working solutions of CIP. All the prepared solutions were kept at 4°C for further use and kept at room temperature before use.

### Extraction Procedure

An aliquot (400 μl) of plasma containing ENR was placed in a 2 ml centrifuge tube. 400 μl of methanol was added to the mixture. The mixture was vortexed for 1 min at high speed and then sonicated for 5 min. After then, the mixture was centrifuged for 10 min at 12,000 rpm and 4°C. The upper, aqueous layer was transferred into an auto-sampler vial through a 0.22 μm microporous membrane using a 1 ml syringe. Plasma extracts were then analyzed for ENR and its metabolite CIP using the described HPLC conditions.

### Chromatographic Conditions

The following chromatographic conditions were used to analyze the plasma extracts: Mobile phase was acetonitrile: 0.1% formic acid = 17:83 (v/v); flow rate: 1 ml/min; excitation wavelength: 278 nm; emission wavelength: 465 nm; injection volume: 20μl; column temperature 30°C; Column: Agilent SB-C18 column (250 × 4.6 mm, 5 um), HPLC under the Agilent 1,290 infinity II separation system.

### HPLC Method Validation

The effectiveness of the HPLC method was validated by evaluating sensibility, specificity, linearity, stability, accuracy, and precision. Eight different blank plasma samples with corresponding standard plasma with ENR standard were evaluated to justify specificity. The stability of plasma samples under three different storage conditions was assessed by determining six replicates of quality control samples (0.1, 1, 5 μg/ml) such as storage at room temperature for 24 h, at 4°C for 24 h, and freeze-thaw cycles (from −20°C to room temperature, three times). Accuracy and precision of intra-day and inter-day were investigated by six replicates of quality control samples on the same day, and for 3 consecutive days, respectively.

### Experimental Animals

All animal experiments were approved by the Animal Administration and Ethics Committee of Lanzhou Institute of Husbandry and Pharmaceutical Sciences, Chinese Academy of Agricultural Sciences. The certificate number was SCXK (Gan) 2019-002. Total 32 healthy pigs (Duroc × Changbai × Dabai) were taken, an average weight of about 27 kg and age 15–16 weeks, randomly divided into four groups, eight pigs in each group. Half female and half male. During the study, the pigs were housed in a clean, quiet environment and fed balanced food, and the water supply was ad-libitum. The average environment temperature and relative humidity were 20°C and 60%, respectively. The test site was GLP/GCP Management Center for Veterinary Drugs, Standard Experimental Animal Field of the Lanzhou Institute of Husbandry and Pharmaceutical Sciences of the Chinese Academy of Agricultural Sciences.

### Administration of Drugs, Blood Sample Collection and Blood Sample Processing

The pigs were kept to adapt to the environment for 7 days before the administration of drugs. The pigs were kept fasted for 12 h, and weight was measured before drug administration. According to the clinical recommendation, a dose of 2.5 mg per kg of body weight was administered once intramuscularly. The blood collection site was the anterior vena cava. Ten percent ENR injection (alkaline) was administered in group-1, 20% ENR injection (acidic) was administered in group-2, 10% ENR injection (Yangkang) was administered in group-3, 20% ENR injection (Nuokang®) was administered in group-4. The blood collection schedule was to be 0 (before administration of drug), 0.083, 0.25, 0.5, 0.75, 1, 1.5, 2, 2.5, 3, 4, 8, 12, 16, 24, 36, 48 60, and 70^th^ h. The blood collection volume was 5 ml. The blood was collected in a heparinized tube and centrifuged at 4,500 rpm for 10 min, and the supernatant was aspirated in another tube. The plasma was kept at −20° C to analyze.

### Data Processing and Statistical Analysis

Linear Trapezoidal with Linear Interpolation calculation method of WINNONLIN noncompartmental analysis program (Version 5.2.1) was used to analyze the experimental data. We obtained the most important pharmacokinetic parameters, that is, C_max_, T_max_ AUC_all_, and T_1/2_ weighting by the 1/Y scheme of the software. The parameters were compared under one-way ANOVA using SPSS software with a 95% confidence interval (CI). The *p*-value of <0.05 was considered statistically significant.

## Results

### Method Validation

**Figure 1:**
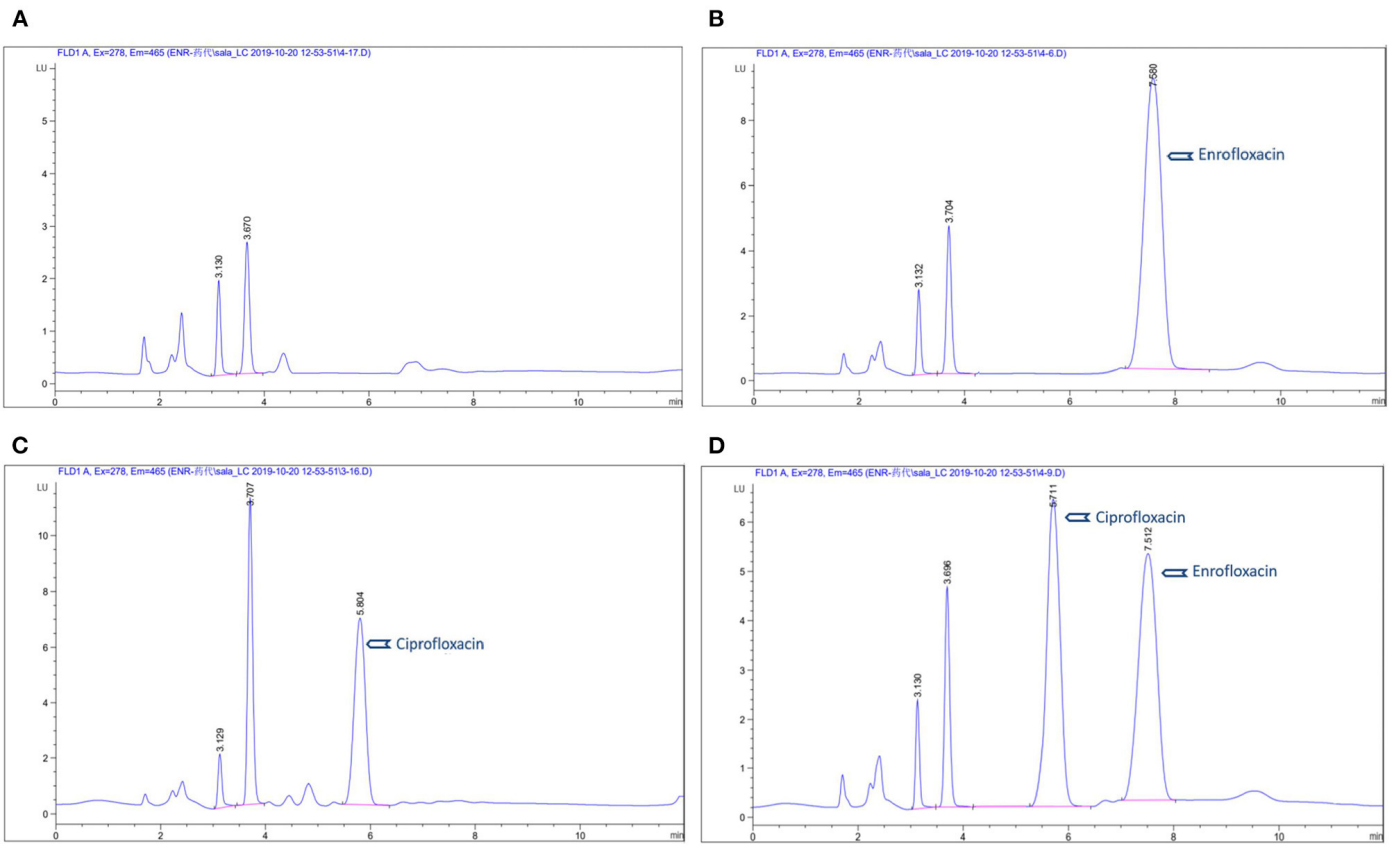
Chromatograms of enrofloxacin in plasma: **(A)** blank control group in plasma; **(B)** chromatograms of ENR in pig plasma; **(C)** chromatograms of ciprofloxacin in standard plasma; **(D)** chromatograms of ENR and ciprofloxacin in standard plasma.

Eight points (0.025, 0.05, 0.1, 0.2, 0.5 1, 2, 5 μg/ml) were considered for establishing a standard curve of enrofloxacin and ciprofloxacin in plasma. Calculations of the standard curve were based on the peak area with the respective concentrations of ENR and CIP. They showed a good selectivity and linear relationship with a correlation coefficient (*R*^2^) = 0.999 in plasma. The mean recovery was more than 94% and 90% for ENR and CIP, respectively. The limit of quantitation (LOQ) was 50 ng/ml in plasma. The intra-day and inter-day precisions (RSD) were <12.7 and <7.3% respectively. The chromatogram (Figure 1) shows **(A)** control blank plasma; **(B)** ENR in pig plasma measured at 7.5 min; **(C)** ciprofloxacin in standard plasma measured at 5.8 min, and **(D)** ENR and ciprofloxacin in standard plasma measured at 7.5 min and 5.7 min respectively which shows the suggested method for the detection of ENR and its active metabolites CIP is specific and accurate.

### Pharmacokinetic Analysis

**Figure 2:**
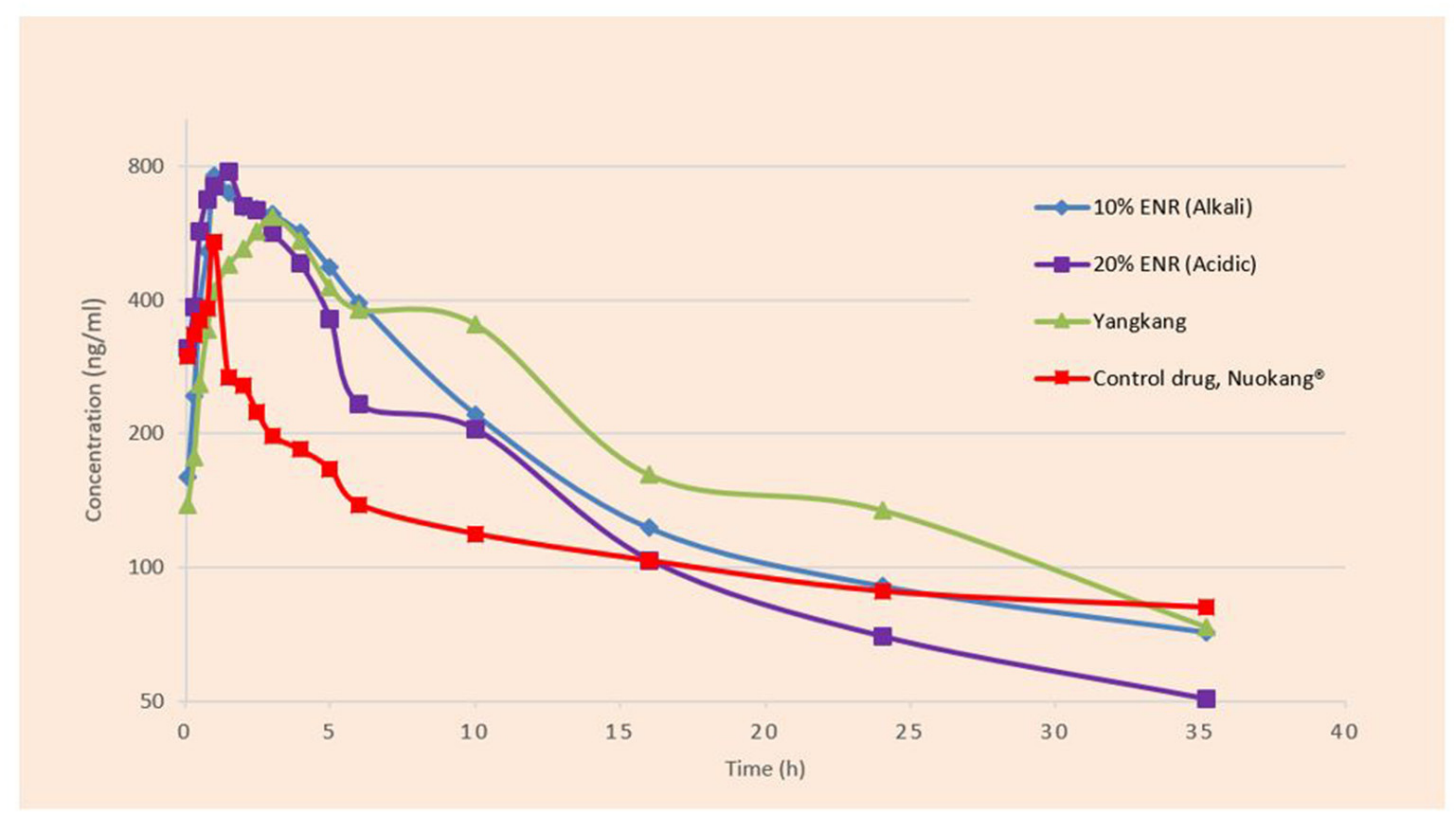
Semi-logarithmic plot of the mean plasma concentration (ng/ml) in pigs at various time points following IM administration of tested and reference formulations of ENR at a single dose of 2.5 mg/kg body weight.

A semi-logarithmic plot of the mean plasma concentration (ng/ml) in pigs at various time points following IM administration of 10% enrofloxacin (Alkali), 20% enrofloxacin (acidic), 10% enrofloxacin (Yangkang), and reference formulations (Nuokang®) at a single dose of 2.5 mg/kg body weight is shown (Figure 2). The main descriptive pharmacokinetic parameters, that is, the maximum plasma concentrations (C_max_), the time to maximum concentration (T_max_), area under the time curve concentration (AUC_all_), and Terminal half-life (T_1/2_) of tested and reference formulations of ENR in pigs are presented in a table (Table 1).

**Table 1:** The main pharmacokinetic parameters of tested and reference formulations of ENR after a single intramuscular administration (2.5 mg/kg body weight) in pigs (Mean ± SD, *n* = 8 in each group).

**Pharmacokinetic parameters**	**10% ENR (Alkali)**	**20% ENR (Acidic)**	**10% ENR (Yangkang)**	**20% ENR (Nuokang^®^) (Reference)**	***P*-values**
T_max_ (h)	2.19 ± 0.0.66^c^	1.50 ± 0.37^b^	2.89 ± 0.24^d^	0.34 ± 0.13^a^	<0.001
C_max_ (ng/ml)	733.84 ± 129.87^ab^	917.00 ± 240.13^b^	694.84 ± 163.49^ab^	621.98 ± 227.25^a^	0.039
AUC_all_ (h*ng/ml)	7754.43 ± 2887.16^b^	8084.11 ± 1543.98^b^	7369.42 ± 2334.99^b^	4194.10 ± 1186.62^a^	0.014
T_1/2_ (h)	10.48 ± 2.72	10.37 ± 2.38	10.20 ± 2.81	10.61 ± 0.86	0.997

a, b, c, d *Different superscripts within a row indicate a significant difference (p < 0.05)*.

### Pharmacodynamic Analysis

The PK/PD indices C_max_/MIC_90_, and AUC/MIC_90_ were calculated (**Table 3**) for the most prevalent pathogens associated with swine diseases, that is, *Streptococcus suis, Haemophilus parasuis, Escherichia coli, Pasteurella multocida, Actinobacillus pleuropneumoniae*, and *Bordetella bronchiseptica* using the mean values of C_max_ and AUC for plasma and the respective minimum inhibitory concentration (MIC) values reported by different authors (Table 2). These data concern the inhibitory activity of various formulations of ENR against some pathogens, causing severe diseases in pigs.

**Table 2:** Minimum inhibitory concentration values for ENR (ng/mL) against respective pathogens associated with swine diseases.

**Microorganism**	**MIC_**50/90**_** **(Nanogram)**	**References**
*Actinobacillus pleuropneumoniae*	≤16/≤16	(29)
*Pasteurella multocida*	≤16/≤16	(29)
*Streptococcus suis*	250/500	(29)
*Haemophilus parasuis*	≤30/1000	(30)
*E. coli*	125/250	(3)
*Bordetella bronchiseptica*	250/500	(31)

## Discussion

Enrofloxacin reveals a concentration-dependent bactericidal activity (26). This drug is very lipophilic and shows amphoteric properties due to the addition of a carboxylic acid and a tertiary amine (32). It is bactericidal and has outstanding activity against both Gram-positive as well as Gram-negative pathogens (33). This antibiotic also can be used to control some intracellular pathogens (34). The extremely poor water solubility and wettability of ENR cause difficulties in the design of pharmaceutical formulations and lead to variable bioavailability (35). In the two formulations, 10% ENR (Alkali) and 20% ENR (Acidic), 2-hydroxypropyl-β-cyclodextrin (HP-β-CD) was used. HP-β-CD is a safe and effective drug carrier commonly used as a cyclodextrin analog. It improves the physicochemical and pharmacokinetic properties of drugs, forming inclusion complexes with drug substrates (36). In 10% ENR (Yangkang**)** formulations, arginine was used for mild alkalinity. In ENR injection, arginine can improve its bacteriostatic function (37). Arginine provides good stability, safety and efficient, long-acting and anti-inflammatory action; maintain certain stability when storing at low-temperature, etc. (38–41).

Comparing with other studies who also administered enrofloxacin @ 2.5 mg/kg body weight as a single dose in pigs, we found almost similar results. In our study, we got T_max_ 2.19, 1.50, 2.89, and 0.34 h when given 10% enrofloxacin (Alkali), 20% enrofloxacin (acidic), 10% enrofloxacin (Yangkang) and reference formulations (Nuokang®) to pigs, respectively which are mostly similar to other studies, that is, 1 h (42), 2 h (43), 1.27 h (3). We obtained C_max_ 733.84, 917.00, 694.84, and 621.98 ng/ml when given 10% enrofloxacin (Alkali), 20% enrofloxacin (acidic), 10% enrofloxacin (Yangkang) and reference formulations (Nuokang®) which are similar to other studies, that is, 694.7 ng/ml (42), 600 ng/ml (43). AUC_all_ were 7,754.43, 8,084.11, 7,369.42, and 4,194.10 ng h/ml for 10% enrofloxacin (Alkali), 20% enrofloxacin (acidic), 10% enrofloxacin (Yangkang) and reference formulations (Nuokang®) to pigs respectively which are similar to another study, that is, 8,903.2 ng h/ml (42). T_1/2_ were 10.48, 10.37, 10.20, and 10.61 h for 10% enrofloxacin (Alkali), 20% enrofloxacin (acidic), 10% enrofloxacin (Yangkang) and reference formulations (Nuokang®) respectively which were longer than the findings of other authors, that is, 6.69 ± 1.71 h (3), 9.3 h (42). We also obtained longer T_1/2_, better C_max_ and AUC_all_ comparing to the studies in other species, that is, sheep (44), goat (45), where ENR at a single dose of 2.5 mg/kg body weight were administered intramuscularly too.

It has been reported that large inter-species differences occur in the half-life of ENR. It depends on the age and development of the liver and kidneys of the host animals (46). Breed or physiological state is also considered for the variability (42). Sometimes ENR converts into ciprofloxacin in the body, which also acts as an antimicrobial agent (45). In this study, we did not find CIP in plasma in contradiction to the findings of other authors who injected 2.5 mg/kg body weight, intramuscularly, for 3 days in older pigs (76–86 kg) (16). It may be due to the younger pigs; we used in our experiment that supports other studies (3, 42). A negligible amount of ciprofloxacin, an active metabolite of enrofloxacin were detected in a study where the authors administered enrofloxacin to younger pigs at a dose of 7.5 mg/kg, subcutaneously (22). The low doses that we administered may be another reason why we didn't find ciprofloxacin. We found longer T_1/2_ in our study compared to other studies. The longer T_1/2_ may interrupt the primary metabolic pathways to metabolize enrofloxacin to ciprofloxacin (22).

Most common pathogens isolated from swine are reported as *Streptococcus suis* (16.9%), *Haemophilus parasuis* (9.7%), *Escherichia coli* (6.3%), *Pasteurella multocida* (3.4%), *Actinobacillus pleuropneumoniae* (0.3%), *Bordetella bronchiseptica* (1.5%), *Salmonella enteria* (2.3%), and *Erysipelothrix rhusiopathiae* (0.9%) (47).

To select the dosage regimens for therapeutic use, three criteria should be satisfied, that is, (a) bacteriological and clinical cure; (b) Least possibility for the strains becoming resistant; (c) No adverse effects on the host (48). Clinically C_max_/MIC for plasma is generally considered for the measurement of treatment efficiency (43). MIC values of the most common gram-negative pathogens are below 60 ng/ml including *Actinobacillus* and *Pasteurella* species, but some species like *Salmonella* and *E. coli* have MIC levels in ranges of 30–125 ng/ml (43). Fluoroquinolone antibacterial agents show concentration-dependent effects, that is, killing rate and killing degree. The killing of bacteria depends on the drug concentration. Pharmacodynamics and Pharmacokinetic properties of fluoroquinolones show the key breakpoint that determines the efficacy of these drugs is C_max_/MIC≥10–12 and AUIC (AUC/MIC) ≥125 (49). This breakpoint also prevents the development of resistant bacteria against fluoroquinolones (50). These findings mainly come from the study of gram-negative bacteria. But recently, researchers evaluated the efficacy of various fluoroquinolones for *Streptococcus pneumoniae* and proposed that the AUC: MIC need to successfully treat Gram-positive bacteria somewhat lower (i.e., 30–50) (51).

MIC_90_ values of enrofloxacin for pathogens in swine ranges from 16 ng/ml to 500 ng/ml even up to 1,000 ng/ml for some resistant pathogens (Table 2). The mean C_max_/MIC and AUC/MIC ratios of all tested and reference formulations of enrofloxacin against *Actinobacillus pleuropneumoniae* and *Pasteurella multocida* showed the breakpoints more than reported values (C_max_/MIC_90_ ≥ 10–12 and AUC/MIC_90_ ≥ 125) indicating that the administration of 2.5mg/kg enrofloxacin of these formulations may have an adequate antibacterial effect and could be considered as an appropriate dose for treatment against these pathogens. But the C_max_/MIC_90_ and AUC/MIC_90_ of all tested and reference formulations of enrofloxacin with the recommended doses were not satisfactory for the key breakpoint against *Haemophilus parasuis, Streptococcus suis, E. coli*, and *Bordetella bronchiseptica* in swine (Table 3).

**Table 3:** The ratios of C_max_/MIC_90_ and AUC/MIC_90_ of the most prevalent pathogens associated with porcine diseases using the mean values of C_max_ and AUC for plasma and the respective minimum inhibitory concentration (MIC_90_) values.

**Antibiotics**	***Actinobacillus*** ***pleuropneumoniae***		***Pasteurella*** ***multocida***		***Streptococcus*** ***suis***		***Haemophilus*** ***parasuis***		***E. coli***		***Bordetella*** ***bronchiseptica***	
	**C_**max**_/** **MIC**	**AUC_**all**_/** **MIC**	**C_**max**_/** **MIC**	**AUC_**all**_/** **MIC**	**C_**max**_/** **MIC**	**AUC_**all**_/** **MIC**	**C_**max**_/** **MIC**	**AUC_**all**_/** **MIC**	**C_**max**_/** **MIC**	**AUC_**all**_/** **MIC**	**C_**max**_/** **MIC**	**AUC_**all**_/** **MIC**
10% ENR (Alkali)	45.87	484.65	45.87	484.65	1.47	15.51	0.73	7.75	2.94	31.02	1.47	15.51
20% ENR (Acidic)	57.31	505.26	57.31	505.26	1.83	16.17	0.92	8.08	3.67	32.34	1.83	16.17
10% ENR (Yangkang)	43.43	460.59	43.43	460.59	1.39	14.74	0.69	7.37	2.78	29.48	1.39	14.74
20% ENR (Nuokang®)	38.87	262.13	38.87	262.13	1.24	8.39	0.62	4.19	2.49	16.78	1.24	8.39

Some manufacturing companies recommend enrofloxacin injections as a single dose of 2.5 mg/kg body weight, that is, Nuokang® produced by Tianjin Zhongsheng Tiaozhan Biotechnology Co., Ltd. China. This drug is known as a long-acting drug. In this experiment, we have used this drug as a reference drug. Another companies ‘Shandong Dezhou Shenniu Pharmaceutical Co., Ltd. China also started to produce three new formulations of enrofloxacin injection with the same dose. After testing all these new formulations and reference formulation, this study ensured longer T_1/2_, indicating a long-term effect, but the obtained C_max_ and AUC were insufficient to kill some microorganisms.

On the other hand, the low antibiotic concentrations can develop antibiotic-resistant bacteria (52) and these bacteria may create side effects on humans and animals (53). The infections caused by resistant organisms are more challenging to treat than infections caused by the non-resistant organism (54). Antibiotic resistance leads to higher treatment costs, prolonged curation, and increased mortality (55). The optimization of the dosage regimen is essential not only for the treatment but also for reducing antimicrobial resistance (26). As enrofloxacin shows a concentration-dependent bactericidal action, and the peak concentration/MIC and AUC/MIC ratios are considered the indicators of efficacy (56), it is essential to optimize the dose regimen by considering both the pharmacokinetics and pharmacodynamics parameters of the tested and reference drugs. The administration of ENR should ensure correspondingly effective concentrations in plasma against the pathogens, causing diseases in swine (44).

The values of PK/PD indices are preliminary used to optimize dosing regimens and dosing intervals on a rational base, followed by validation in clinical studies for systemically acting antimicrobial agents (57). A valued dosing strategy for infectious diseases requires a thorough understanding of the complex connections among germs, drugs, and the immune system of the host (58). Fluoroquinolone antibiotics have a rapid bactericidal effect and show a significant post-antibiotic impact (59). Post-antibiotic effects also affect dosing strategies (60). The existence of post-antibiotic effects, that is, the inhibition of bacterial growth after limited exposure of microorganisms to antibiotics (61), can improve the therapeutic effect when the marginal pharmacokinetic/pharmacodynamic index value is high and can extend the dosing interval (60). Factors that influence the accuracy of efficacy predictions based on PK/PD indices are related to inoculation effect, microbial growth rate (generation time), the growth phase of an invading organism, the response of the host to the pathogen (immune system, drug diffusivity, pH at the site of the infection), infection site (exudate nature, tissue perfusion, natural barriers), etc. (62). For this reason, the calculated ratios are surrogate markers of efficacy. Further studies in a clinical context would be necessary to evaluate the results obtained in this research.

## Conclusion

The pharmacokinetic parameters showed the tested formulations 10% enrofloxacin (Alkali), 20% enrofloxacin (Acidic), and 10% enrofloxacin (Yangkang) is somewhat better comparing to the reference formulation 20% enrofloxacin (Nuokang®) in the swine model. All tested and reference formulations of ENR, administered at a single dose of 2.5 mg/kg IM, could be used to treat swine diseases caused by *Actinobacillus pleuropneumoniae* and *Pasteurella multocida*. But the dose of these formulations would not be effective against some important pathogens like *Haemophilus parasuis, Streptococcus suis, E. coli*, and *Bordetella bronchiseptica* because the C_max_/MIC_90_ values were nearly 3–16 times lower than 10 and the AUC/MIC_90_ values were nearly 4–30 times lower than 125. It can be concluded that the dose of both tested and reference formulations was not sufficient to treat the pigs infected by the pathogens having more MIC_90_ scores. Further studies are required to optimize the dosage regimen and establish the safety of the dosage of tested and reference formulations in clinical applications.

## Data Availability Statement

The raw data supporting the conclusions of this article will be made available by the authors, without undue reservation.

## Ethics Statement

The animal study was reviewed and approved by the Animal Administration and Ethics Committee of Lanzhou Institute of Husbandry and Pharmaceutical Sciences, Chinese Academy of Agricultural Sciences. The certificate number was SCXK (Gan) 2019-002.

## Author Contributions

JZ and SAh: conceptualization. SAh and JS: methodology and validation. FC: investigation. SAr and JS: resources. SAh: writing—original draft preparation. JZ, BL, and XZ: supervision. JZ: funding acquisition. All authors contributed to the article and approved the submitted version.

## Conflict of Interest

The authors declare that the research was conducted in the absence of any commercial or financial relationships that could be construed as a potential conflict of interest.
